# A rare case of concomittant pancreatic adenosquamous and neuroendocrine tumours

**DOI:** 10.1093/jscr/rjac377

**Published:** 2022-08-17

**Authors:** Izziddine Vial, Ambareen Kausar

**Affiliations:** Department of General Surgery, East Lancashire NHS Trust, Royal Blackburn Hospital, Blackburn, UK; Department of General Surgery, East Lancashire NHS Trust, Royal Blackburn Hospital, Blackburn, UK

**Keywords:** pancreatic tumours, synchronous tumours, adenosquamous, NET tumour

## Abstract

Only two cases of concomitantly occurring adenosquamous and neuroendocrine tumours (NET) have been reported in the literature. We report a case where both NET and adenosquamous are simultaneously occurring. A 42-year-old lady was hospitalized following painless jaundice and loss of weight. Computer tomography scan showed 3.0 x 2.9 cm hypo enhancing and hypovascular mass in the uncinate/head process. Another hypervascular mass was seen in the body of the pancreas. Pathological examination showed that the lesion in the uncinate process/neck of pancreas was an adenosquamous in the main pancreatic duct intraductal papillary mucinous neoplasm. The second tumour was a NET measuring 36 mm with no metastatic involvement. These findings suggested concurrently occurring but separated adenocarcinoma and NET tumours. This is the third case in the literature where both NET and adenosquamous are happening concomitantly, and the previous two other cases are also reviewed in the article.

## INTRODUCTION

A patient presenting double-primary tumour in the pancreas is rare. Most case reports have described double-pancreatic tumours where a single tumour is of mixed histology such as adenoneuroendocrine or where a pancreatic ductal adenocarcinoma is either occurring simultaneously or derived from an intraductal papillary mucinous neoplasm (IPMN) or there is synchronous pancreatic neuroendocrine tumour (NET) and IPMN [1–5]. Concomitant adenocarcinoma and NET in the pancreas is extremely uncommon [6, 7]. We report our management experience of a pre-operatively detected adenocarcinoma involving the head of the pancreas and a NET involving the body of the pancreas.

## CASE PRESENTATION

A 42-year old lady presented to the accident and emergency department with painless jaundice (both eyes and skin), loss of appetite, itching, pale stool, dark urine, lethargy, nausea and significant weight loss which started 3–4 months ago (lost around 20 kg overall). On examination, her abdomen was soft, non-tender but distended and clinically jaundiced. Her past medical history consisted of Type 2 Diabetes Mellitus, dyspepsia, migraine and hypertension.

## INVESTIGATIONS

Her initial blood tests showed normal full blood count and renal function tests. However, her liver function tests were deranged showing an obstructive picture. Bilirubin was 100 μmol/L, alanine transaminase was 452 μmol/L, alkaline phosphatase was 616 μmol/L and gamma-glutamyl transpeptidase was 1155 iu/L. Amylase was normal at 53 μmol/L with a slightly elevated c-reactive protein of 12. Her carbohydrate antigen 19-9 was 252 kU/L.

Her hepatitis and human immunodeficiency virus screen came back as normal.

The computed tomography (CT) triple phase scan showed 3.0 x 2.9 cm hypo enhancing and hypovascular mass in the uncinate/head process. This was causing mild biliary and pancreatic ductal dilatation. A separate hypervascular mass was seen in the body of the pancreas measuring 3 cm causing pancreatic duct dilatation and gland atrophy.

The magnetic resonance imaging (MRI) pancreas and magnetic resonance cholangiopancreatography (MRCP) showed the same findings as of the CT triple phase scan. However, the tumours could be further characterized. The mass within the uncinate process has imaging features more in keeping with pancreatic adenocarcinoma while the mass in the pancreatic body was more difficult to characterize but a NET was more likely.

The CT thorax was completely normal showing no metastatic disease.

**Table 1:** Literature describing synchronous tumours in the pancreas

Author & year	Age/Sex	Preoperative diagnosis	Surgery	Adenocarcinoma		NET		Postoperative outcome
				Location, size (mm)	Pathology	Location, size (mm)	Pathology	
Sastry *et al*. 2014 [7]	81/Male	Not described	Pancreatoduodenectomy	1. Periampullary, 35 2. Uncinate process, 31	1. Poorly differentiated adenosquamous with lymph nodes invasion 2. Moderately differentiated with peripancreatic fat invasion	Head, 12	Well differentiated with cystic degeneration	Died, 10 months
Liu *et al*. 2020 [11]	74/Male	IPMN	Total pancreatectomy and splenectomy	Body, 40	Ductal adenocarcinoma	Tail, 6	-	10 months, no signs of relapse

## DIFFERENTIAL DIAGNOSIS

We had a patient with two distinct masses in their pancreas. After the complimentary radiological investigations, our differential diagnosis for the tumour in the uncinate process was adenocarcinoma while our diagnosis hypothesis for the tumour in the pancreatic body either a NET or a solid pseudo papillary epithelial neoplasm or a malignant IPMN.

## TREATMENT

After discussion at the hepatobiliary multidisciplinary team meeting, it was decided to go straight for surgery—in keeping with the patient’s wishes. The patient was consented for a total pancreatectomy with preservation of the spleen.

An initial exploratory laparotomy was performed where no evident metastasis was seen. During the surgery, two tumours were clearly identified. There was also a large lymph node on top of the right hepatic artery around the superior mesenteric artery that proceeded behind the porta hepatitis up to the coeliac axis. This was removed and sent to histopathology. A pylorus preserving pancreatoduodenectomy along with total pancreatectomy was performed. For reconstruction, an end to side hepato-jejunostomy was performed and antecolic duodeno-jejunostomy on a single loop.

## OUTCOME AND FOLLOW-UP

The patient recovered on the intensive care unit for 4 days and was cared for 13 days on the ward before being discharged home. It took some time to get her diabetes under control.

Histological analysis showed that the lesion in the uncinate process/neck of pancreas was an adenocarcinoma in the main pancreatic duct IPMN. There was focal squamous differentiation with some areas of clear cell appearance and there was mucinous secretion. It was 32 mm in maximum diameter and a Grade 2. There was peri-neural invasion and it infiltrated the peri-pancreatic fatty tissue, duodenal muscularis propria and mucosa as well as lymphovascular invasion. The large lymph node that was removed separately showed a metastatic focus in the sub-capsular focus. All transection margins were clear albeit it was only <1 mm from the anterior surface. Fourteen of the 15 nodes showed metastatic adenocarcinoma and there was background IPMN branch main duct focus. The tumour was graded as Grade 2 pT2 pN2 Mx R0.

The second tumour was in the body. It was a circumscribed neuroendocrine tumour 36 mm in diameter and appeared to infiltrate the capsule at some sections. There was lymphovascular invasion and the Mib-1 index was 1% making it a grade 1 neuroendocrine tumour. It was limited to the pancreas and was T2 with no peri-neural infiltration. Five lymph nodes around this tumour showed no metastatic NET tumour. This was also thought to be an R0 tumour. There was some adjacent cell hyperplasia. The tumour was graded as Grade 1 pT2 NET No Mx R0.

After surgery, she completed 6 cycles adjuvant chemotherapy with FOLFIRNOX. Unfortunately, 4 months later, a follow-up CT scan showed a large tumour recurrence. Enhancement pattern suggested it to be secondary to the adenocarcinoma with squamous cell differentiation rather than a NET. The tumour recurrence was seen within the portacaval space encasing the right hepatic artery extending into the small bowel mesentery and also involving the superior mesenteric artery and superior mesenteric vein. Enlarged lymph nodes were seen within the small bowel mesentery. The CA 19-9 had also risen to 191 from 37 previously.

## DISCUSSION

Synchronous primary tumours of the pancreas are extremely rare. The incidence of having concomitant adenocarcinoma and NET is even rarer [7, 8]. The occurrence of numerous primary malignancies in patients with pancreatic NET is high in contract to the general population. Pancreatic NET is correlated with numerous types of other tumours. This may happen because of accumulated growth stimulation by the secreted hormones or a genetic alteration that gives rise to tumorigenesis [9]. However, the physiology linking NET and adenocarcinoma is still not understood and further research need to be done.

Usually, synchronous tumours of the pancreas are found in previously diseased pancreases with chronic pancreatitis or associated with premalignant lesions such as IPMN as presented in the case above. It is well known that there is relationship between adenocarcinoma and IPMN. Yamaguchi *et al*. [10] reported that ~20% of patients from a series of 765 patients who underwent surgery for IPMN also had pancreatic ductal adenocarcinoma which were either derived from IPMN or synchronous with IPMN.

A PubMed database search found just two cases with concomitant adenocarcinoma and NET (Table 1). Considering our case, there are just three cases reported (two males and one female, mean age 65.7 years old) described in the literature. Sastry *et al*. reported a case of three concomitant tumours of the pancreas: an adenosquamous, an adenocarcinoma and a NET. The adenocarcinoma and NET were incidental tumours found during the histologic process. However, Liu *et al*. were able to identify both the NET and the adenocarcinoma as well as a pseudocyst pre-operatively.

In conclusion, the possibility of multiple primary tumours has to be considered when managing multiple tumours in the pancreas. The use of optimal imaging modalities allows for optimum preoperative diagnosis and planning, which result in the most appropriate treatment option available to the patient.
